# The Road Less Traveled? Unconventional Protein Secretion at Parasite–Host Interfaces

**DOI:** 10.3389/fcell.2021.662711

**Published:** 2021-05-24

**Authors:** Erina A. Balmer, Carmen Faso

**Affiliations:** Institute of Cell Biology, University of Bern, Bern, Switzerland

**Keywords:** unconventional secretion, protist parasites, host–pathogen interface, moonlighting, glycolysis

## Abstract

Protein secretion in eukaryotic cells is a well-studied process, which has been known for decades and is dealt with by any standard cell biology textbook. However, over the past 20 years, several studies led to the realization that protein secretion as a process might not be as uniform among different cargos as once thought. While in classic canonical secretion proteins carry a signal sequence, the secretory or surface proteome of several organisms demonstrated a lack of such signals in several secreted proteins. Other proteins were found to indeed carry a leader sequence, but simply circumvent the Golgi apparatus, which in canonical secretion is generally responsible for the modification and sorting of secretory proteins after their passage through the endoplasmic reticulum (ER). These alternative mechanisms of protein translocation to, or across, the plasma membrane were collectively termed “unconventional protein secretion” (UPS). To date, many research groups have studied UPS in their respective model organism of choice, with surprising reports on the proportion of unconventionally secreted proteins and their crucial roles for the cell and survival of the organism. Involved in processes such as immune responses and cell proliferation, and including far more different cargo proteins in different organisms than anyone had expected, unconventional secretion does not seem so unconventional after all. Alongside mammalian cells, much work on this topic has been done on protist parasites, including genera *Leishmania*, *Trypanosoma*, *Plasmodium*, *Trichomonas*, *Giardia*, and *Entamoeba*. Studies on protein secretion have mainly focused on parasite-derived virulence factors as a main source of pathogenicity for hosts. Given their need to secrete a variety of substrates, which may not be compatible with canonical secretion pathways, the study of mechanisms for alternative secretion pathways is particularly interesting in protist parasites. In this review, we provide an overview on the current status of knowledge on UPS in parasitic protists preceded by a brief overview of UPS in the mammalian cell model with a focus on IL-1β and FGF-2 as paradigmatic UPS substrates.

## Introduction

A protein destined to be secreted carries a signal peptide or signal leader sequence and is often transported from the ER to the Golgi apparatus, to the trans-Golgi network for sorting, and finally to the plasma membrane via vesicular carriers (Palade, 1975; Rothman, 1994). This is a classic dogma of the cell biology of secretory protein trafficking and remains true for many proteins leaving the intracellular space. However, more and more leaderless proteins are identified in the secretomes and surface proteomes of various species. These proteins reach the plasma membrane while bypassing classical secretory routes. Furthermore, some proteins with leader sequences were discovered to pass through the ER but to bypass the Golgi apparatus on their way to the plasma membrane. These non-canonical secretion pathways are collectively summarized under the term “unconventional protein secretion” (UPS) to distinguish them from classic canonical secretion following the ER–Golgi pathway (Rabouille et al., 2012).

**FIGURE 1:**
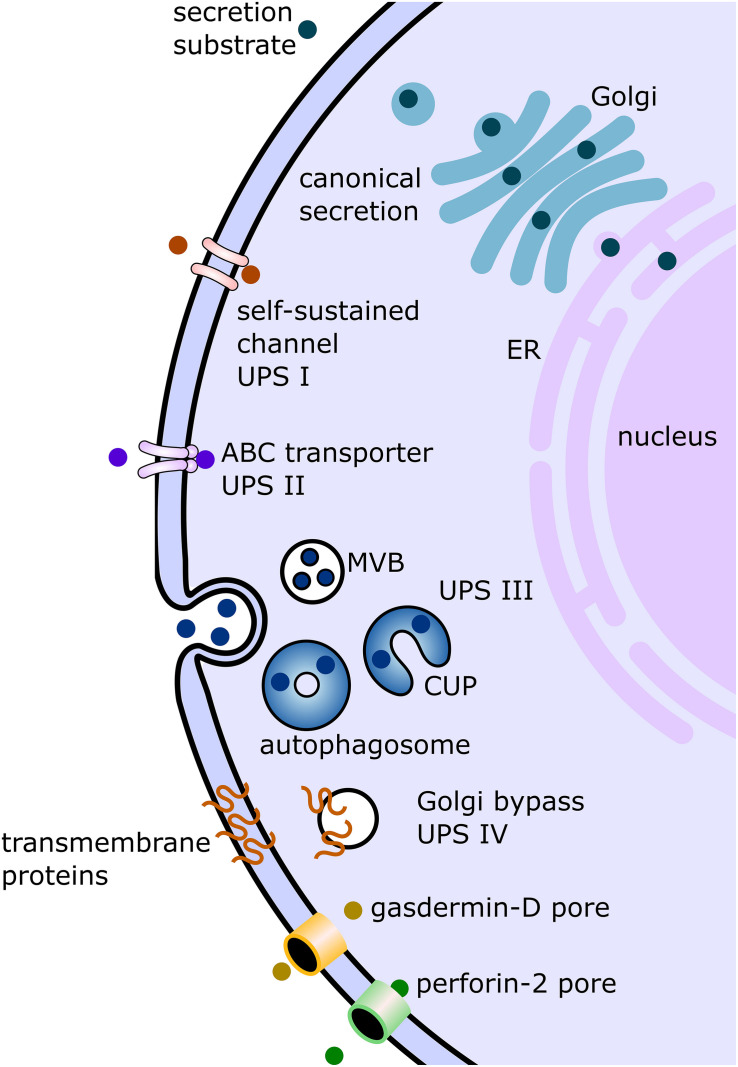
Displayed are the canonical conventional as well as unconventional secretion pathways described in studies on mammalian cells and/or studies on protist parasites. In the conventional canonical secretion pathway, the secreted substrate passes through the endoplasmic reticulum (ER) to the Golgi apparatus (Golgi) and then to the plasma membrane via vesicular carriers. Several non-canonical pathways have been discovered and summarized under the term “unconventional protein secretion” (UPS). There are four pathway types formally described. UPS pathway I (UPS I) consists of substrates without a canonical signal sequence, transported via self-sustained channels. The best characterized substrate of this pathway is fibroblast growth factor. UPS route II is mediated by ABC transporters, as in the secretion of the leaderless Leishmania HASPB. UPS pathway III (UPS III) was mainly studied in mammalian cells under starvation conditions. In this pathway, leaderless substrates such as IL-1b are transported across the plasma membrane involving vesicular compartments for UPS (CUPS) which turn into autophagosomes. These autophagosomes then either fuse with multivesicular bodies (MVB) or fuse directly with the plasma membrane. The fourth described pathway (UPS IV) involves transmembrane proteins sequence for secretion, but the substrate (for example, cystic fibrosis transmembrane conductance regulator) bypasses the Golgi apparatus on the way from the ER to the plasma membrane. The two additional unconventional secretion pathways depicted in this figure involve the formation of pores as in UPS I, but these pores are not self-sustained and involve gasdermin-D (in case of IL-1b secretion) or perforin-2 facilitation (in case of IL-33).

Currently, there are mainly four described eukaryotic UPS pathways (Figure 1 and Table 1). UPS type I is probably the best characterized and was mainly elucidated using fibroblast growth factor 2 (FGF2) as its model substrate (Wegehingel et al., 2008; La Venuta et al., 2015; Steringer et al., 2015; Legrand et al., 2020). In this secretion pathway, the leaderless substrate is translocated to the extracellular space with self-sustained formation of pores in the plasma membrane. The second described UPS pathway, type II, is mediated by ABC transporters, which transport leaderless acylated proteins across the plasma membrane. Although there are limited reports investigating the mechanisms of this pathway in-depth, several eukaryotes appear to employ this mode of secretion [mammals (Flieger et al., 2003), *Drosophila* (Ricardo and Lehmann, 2009), yeast (McGrath and Varshavsky, 1989), *Leishmania* (Denny et al., 2000; Stegmayer et al., 2005; MacLean et al., 2012), and *Plasmodium* (Möskes et al., 2004)]. This suggests that ABC transporter-mediated non-conventional secretion is conserved among eukaryotes (Ricardo and Lehmann, 2009). UPS type III in mammalian cells involves intracellular vesicular structures induced upon starvation (Bruns et al., 2011; Malhotra, 2013; Cruz-Garcia et al., 2020). As shown for IL-1β (Rubartelli et al., 1990; Andrei et al., 2004; Qu et al., 2007), AcbA in *Dictyostelium discoideum* (Kinseth et al., 2007; Cabral et al., 2010) and its yeast ortholog Acb1 (Duran et al., 2010; Manjithaya et al., 2010; Curwin et al., 2016) compartments (CUPS compartments for UPS, endosomes or exosome-like compartments) transport leaderless cargo proteins across the plasma membrane (Nickel and Rabouille, 2009; Curwin et al., 2016). UPS route type IV is the only known UPS pathway, which includes substrates presenting a leader sequence. These substrates are trafficked through the ER, but bypass the Golgi on their way to the plasma membrane (Gee et al., 2018; Kim et al., 2018). Even though not much is known about the machinery mediating the Golgi bypass, the best studied substrate of this pathway is the cystic fibrosis transmembrane conductance regulator (CFTR; Yoo et al., 2002; Gee et al., 2011; Rabouille et al., 2012). CFTR travels via the so-called pericentrosomal intermediate compartment, where it can reach recycling endosomes and is then transported to the plasma membrane (Marie et al., 2009; Prydz et al., 2012; Rabouille et al., 2012). Although the current UPS landscape appears complex, it is probably that more UPS routes remain to be discovered. Furthermore, it is also probable that the same substrate can be transported over several different routes, depending on cellular conditions.

**TABLE 1:** Overview of UPS pathways including information on known machineries or components and relevant cargo examples.

**UPS route type**	**Signal sequence for secretion**	**Known machineries/components**	**Examples of known cargo**	**Selected references**
Type I	No	Self-sustained channels (binding to α1-subunit of Na, K-ATPase and PI(4,5)P_2_)	FGF2, HIV-TAT	Temmerman et al., 2008; Zeitler et al., 2015; Legrand et al., 2020
Type II	No	ABC- transporters, N-terminal domain	HASPB, PfAK2	Denny et al., 2000; Stegmayer et al., 2005; MacLean et al., 2012; Thavayogarajah et al., 2015; Kim et al., 2018
Type III	No	Vesicles, inflammasome, heat shock protein, GRASP, ESCRTs	**IL-1β**, Acb1, HMGB1	Nickel and Rabouille, 2009; Dupont et al., 2011; Curwin et al., 2016; Cruz-Garcia et al., 2020; Kim et al., 2020
Type IV	Yes	Golgi bypass via pericentrosomal intermediate compartment, transmembrane domain	CFTR	Rabouille et al., 2012; Gee et al., 2018; Kim et al., 2018
Pore formation	No	Gasdermin D, perforin-2	**IL-1β**, IL-33	Martín-Sánchez et al., 2016; Heilig et al., 2017; Lieberman et al., 2019; Hung et al., 2020

*IL-1β is highlighted to emphasize its known use of two different UPS pathways. FGF2, fibroblast growth factor 2; HASPB, hydrophilic acylated surface protein B; GRASP, Golgi reassembly stacking protein; PI(4,5)P_2_, phosphatidylinositol-4,5-bisphosphate; CFTR cystic fibrosis transmembrane conductance regulator; PfAK2, *Plasmodium falciparum* adenylate kinase 2; ESCRT, endosomal sorting complex required for transport.*

With more and more published secretomes and surface proteomes and therein found leaderless proteins, the interest in studies on UPS is increasing. Involved in the most central mechanisms of disease such as inflammation and cancer cell proliferation and, specifically for this review, secretion of virulence factors in parasites, it is of crucial importance to understand the underlying mechanisms of unconventional secretion. Unraveling machineries involved in UPS will severely impact translational research and the development of therapeutic strategies in associated diseases, which are often caused by aberrant UPS-related processes (Kim et al., 2018).

In this short review, we will highlight the similarities and differences in UPS pathways and substrates in protist parasites and mammalian cells. Our objective is to provide the reader with a solid overview of developments in this fast growing, but still mostly underexplored, field of research.

## Unconventional Protein Secretion in Mammalian Cells

### Secretion of Interleukin IL-1β

The roots of UPS research lie over 30 years back in time, in studies carried out primarily on mammalian cells. When the human cytokine interleukin 1 was sequenced, it was found to lack a leader sequence allowing this secreted molecule to enter the ER–Golgi pathway for secretion (Auron et al., 1984). The non-canonical secretion of cytokines has been studied since, often using interleukin 1 beta (IL-1β) as a model substrate (Rubartelli et al., 1990; Andrei et al., 2004; Daniels and Brough, 2017). IL-1β is a crucial cytokine in immune reactions and has been associated with many different states of disease, infection, and injury (Dinarello, 1996; Lopez-Castejon and Brough, 2011). The precursor form pre-IL-1β is converted to the active cytokine *via* the inflammasome apparatus (Dupont et al., 2011; Lamkanfi, 2011; Lamkanfi and Dixit, 2012). Although much effort has been invested in elucidating secretion mechanisms for IL-1β, they are not yet fully understood (Lopez-Castejon and Brough, 2011).

Several different pathways were suggested by which active IL-1β is translocated across the cell membrane. The release of IL-1β is induced by a variety of secretion stimuli: pathogen-associated molecular patterns (PAMPs) or danger-associated molecular patterns (DAMPs; Takeuchi and Akira, 2010; Lopez-Castejon and Brough, 2011), depending on cell type and stimulus (Lopez-Castejon and Brough, 2011; Kim et al., 2018). One mechanism involves vesicular carriers as part of a UPS type III route for IL-1β release (Rabouille et al., 2012). As described in Dupont et al. (2011), IL-1β is captured by the autophagy-related process of sequestration of cytosol into a vesicular organelle called the autophagosome, which transports IL-1β to the plasma membrane for secretion. Some studies suggest that instead of directly fusing with the plasma membrane, the autophagosome fuses with a multivesicular body (MVB) to form an “amphisome” before fusing with the plasma membrane (Zhang et al., 2015). Even though not completely elucidated, the availability of Golgi reassembly stacking protein (GRASP) seems to be of great importance for successful translocation of IL-1β, since the knockdown of GRASP leads to strongly decreased secretion (Nickel and Rabouille, 2009; Dupont et al., 2011). Induction of autophagy appears to enhance inflammasome activity and, therefore, the production of active IL-1β. This suggests a strong link between autophagy and cytokine secretion, which was shown to influence severity of inflammation (Dupont et al., 2011). Zhang et al. (2015) investigated the translocation process of IL-1β into the autophagosomal compartment for UPS (CUPS) and found a strong dependency on a heat shock protein (HSP90) and GRASP proteins (Zhang et al., 2015). Additionally, Zhang et al. (2015) suggested in the same study that mature IL-1β targets a vesicular structure, which precedes sequestration into the autophagosome. This preceding step was now studied in greater detail (Zhang et al., 2020). This study suggests that the described vesicular structure corresponds to the ER–Golgi intermediate compartment (ERGIC), and IL-1β is transported into this compartment via a TMED10-mediated protein channel (Zhang et al., 2020).

Similar to IL-1β, AcbA (acylcoenzyme A-binding protein) and its yeast ortholog Acb1 have been shown to traffic along the UPS type III route (Kinseth et al., 2007; Cabral et al., 2010; Duran et al., 2010; Manjithaya et al., 2010; Curwin et al., 2016). In yeast, Acb1 was found to be secreted during starvation of the cell, despite the lack of a signal sequence for secretion (Duran et al., 2010; Curwin et al., 2016). The machinery involving transport of the cargo via CUPS requires ESCRT proteins I, II, and III for successful secretion of Acb1 (Duran et al., 2010; Curwin et al., 2016). As ESCRT proteins are necessary to build up an MVB, it was hypothesized that UPS III involves secretion via MVBs (Duran et al., 2010). The results of an in-depth study by Curwin et al. (2016) found that even though certain components for the formation of a classic MVB are present, a part of the necessary machinery is lacking (ESCRT-0 or Vps4 proteins; Curwin et al., 2016). The authors suggest that instead of canonical MVBs, a specific doubled membrane-bounded multivesicular compartment is involved in Acb1 secretion (Curwin et al., 2016). They speculate that this compartment might be the functional ortholog of the amphisomes in IL-1β secretion (Zhang et al., 2015; Curwin et al., 2016). Studies on AcbA in *Dictyostelium* found that secretion involves cortical vesicles, which fuse with the surface membrane, or exocytosis of the vesicles from MVBs in a GRASP protein-dependent fashion (Kinseth et al., 2007; Cabral et al., 2010).

As Kim et al. (2020), showed in a recent study, the secretion of high-mobility group box 1 protein (HMGB1) represents another cargo of UPS pathway III. This protein, which is usually found in the nucleus where it acts as a DNA chaperone, has the function of a danger-associated molecular pattern when transported to the extracellular space and induces inflammation and cell migration (Andersson and Tracey, 2011). For its transport across the plasma membrane, a similar vesicular trafficking machinery is involved as has been found in IL-1b, including GRASP (GORASP2/GRASP55) and heat shock protein (HSP90AA1; Dupont et al., 2011; Zhang et al., 2015; Kim et al., 2020).

Another mode of IL-1β secretion does not involve vesicular carriers but the formation of pores in the plasma membrane (Martín-Sánchez et al., 2016; Heilig et al., 2017). IL-1β is also secreted by pyroptotic cells about to undergo inflammasome-controlled programmed cell death (Fink and Cookson, 2005; Lamkanfi and Dixit, 2012). The secretion of IL-1β by macrophage cells, seems to depend strongly on permeabilization of the plasma membrane (Martín-Sánchez et al., 2016). Gasdermin D has long been known as a regulator of pyroptosis, forming pores in the plasma membrane of a cell to induce programmed cell death (Ding et al., 2016). It was found that IL-1β secretion strongly depends on gasdermin Ds’ pore-formation ability (He et al., 2015; Martín-Sánchez et al., 2016). However, whether IL-1β is secreted passively due to cell death, or actively by living cells, is a question that was only recently answered (Evavold et al., 2018; Kuriakose and Kanneganti, 2018; Lieberman et al., 2019). In a study by Evavold et al. (2018), gasdermin D pores were shown to be necessary for IL-1β transport across an intact plasma membrane of living macrophages. This suggests that gasdermin D is not only responsible for passive interleukin secretion during pyroptosis but also for its active transport in living cells (Heilig et al., 2017; Evavold et al., 2018; Lieberman et al., 2019). Even though this mode of secretion via pores has similarities with the UPS type I route, which was mainly studied in FGF2 (elaborated on in the section “Translocation and Binding of Fibroblast Growth Factor 2”), neither pro-IL-1β nor mature IL-1β was found to bind to membranes (Martín-Sánchez et al., 2016); hence, the transport is not mediated by the substrate itself. In this respect, this route does not fully match the description of UPS route type I and rather represents a separate UPS route. It is very likely that also other cargos employ more than one route of unconventional secretion or show secretion mechanisms, which do not fit in the previously described four routes of unconventional secretion as for IL-1β. Elucidating the mechanisms of non-canonical secretion of IL-1β boosts translational research on this molecule, which has shown to have not only positive effects as a mediator in immune response but, if found in elevated levels, can also have severe negative effects in conditions such as diabetes type II (Maedler et al., 2009).

Importantly, interleukin secretion in mammalian cells also plays a crucial role in the innate immune reaction against parasitic infections (Ryan et al., 2020). Interleukin 33 (IL-33) does not have a signal sequence, while it is still found to be secreted early on when a parasite is invading, involved in priming and/or modulating adaptive immune response of the host (Cayrol and Girard, 2018; Ryan et al., 2020). Similar to one of the pathways for IL-1β, IL-33 secretion has been brought into connection with cell death, while for both interleukins, cell death seems not to be a strict requirement for their release (Kouzaki et al., 2011; Chen et al., 2015; Molofsky et al., 2015; Daniels and Brough, 2017; Ryan et al., 2020). As Hung et al. (2020) could show in a recent study, in mouse dendritic cells, IL-33 export was facilitated by perforin-2, which forms pores in the plasma membrane, similar to gasdermin D.

Interleukin 33 has been shown to have a significant impact on different T cells, influencing their differentiation, behavior, and physiology (Alvarez et al., 2019; Ryan et al., 2020). This makes this interleukin an attractive potential target for translational research as an immunotherapy treatment in toxoplasmosis, malaria, and helminth infections (Jones et al., 2010; Yasuoka et al., 2011; Fairlie-Clarke et al., 2018; Ryan et al., 2020).

### Translocation and Binding of Fibroblast Growth Factor 2

Fibroblast growth factor 2 (FGF2) is the substrate that quickly became the most studied UPS cargo in mammalian cells. The mechanisms by which it is secreted are by far the most elucidated and have been allocated to UPS route type I. FGF2 is an angiogenic mitogen with a wide variety of functions, from cell growth and tissue repair to differentiation of the nervous system. While its extracellular form shows pro-angiogenic and pro-inflammatory activity, FGF2 acts as a mitogenic stimulus when localized in the nucleus (Bikfalvi et al., 1997; Jeffery, 1999; Collingridge et al., 2010; Gómez-Arreaza et al., 2014; Yoon et al., 2018), thus, making FGF-2 an example of “moonlighting” protein, a protein with multiple functions (Jeffery, 2018). FGF2 was shown to be secreted directly and independently of ATP (Schäfer et al., 2004). The current state of knowledge concerning FGF2 secretion involves recruitment from the cytosol to the inner leaflet of the plasma membrane where it binds to the α1-subunit of Na, K-ATPase and phosphatidylinositol-4,5-bisphosphate [PI(4,5)P_2_] (Temmerman et al., 2008; Legrand et al., 2020). As Legrand et al. (2020) hypothesized in a recent study (Legrand et al., 2020), the α1-subunit of the Na, K-ATPase would accumulate FGF2 at the inner plasma membrane leaflet and, thus, facilitate PI(4,5)P_2_-dependent secretion to the cell surface. When reaching the extracellular space, FGF2 seems to bind immediately to cell surface heparan sulfate proteoglycans (HSPGs), which are anchored in the plasma membrane and trap secreted FGF2 (Zehe et al., 2006; Wegehingel et al., 2008). The binding of FGF2 to HSPG serves a storage function as well as the protection of FGF2 against degradation and denaturation (Flaumenhaft et al., 1990; Coltrini et al., 1993). As Wegehingel et al. (2008) could show, FGF-2 is no longer functional if it is modified with a leader sequence to enter the canonical secretion pathway. The addition of a leader sequence leads to posttranslational modifications, which prevent FGF-2 from binding to heparan sulfate proteoglycans and, therefore, inhibits storage and signal transduction (Wegehingel et al., 2008). Secretion of HIV-TAT, an HIV-1 protein, which acts extracellularly as a viral toxin, has been suggested to use the same UPS core mechanism as FGF-2 (Zeitler et al., 2015). Here, too, oligomerization involving PI(4,5)P_2_, led to the formation of plasma membrane pores through which HIV-TAT reached the extracellular space (Zeitler et al., 2015).

### Significance of Unconventional Protein Secretion in Mammalian Cells

With IL-1β and FGF2 being the most studied, there are many other substrates in mammalian cells that were identified to be secreted unconventionally (Nickel, 2005; Kim et al., 2018). Galectins and annexins (Hughes, 1999; Popa et al., 2018), other interleukins such as IL-1α and IL-33 (Daniels and Brough, 2017), transmembrane proteins CFTR and Mpl (Gee et al., 2011; Cleyrat et al., 2014; Kim et al., 2016, 2018; Noh et al., 2018), and the multifunctional glycolytic enzyme GAPDH, which was found to be associated with exosomes (Théry et al., 2009; Mathivanan et al., 2010; Chauhan et al., 2015), represent a few examples. Studies performed on neuronal cell lines show that hundreds of neuronal surface proteins reach the plasma membrane in a UPS pathway bypassing the Golgi apparatus (UPS type IV; Hanus et al., 2016). All these cases in various cell types emphasize the significance of UPS in mammalian cell biology. The mechanisms by which these substrates are secreted are only partly understood. In mammalian cell model systems, mechanisms for all described UPS routes are suggested, depending on the cargo and secreting cell type (Evavold et al., 2018; Kim et al., 2018; Noh et al., 2018; Popa et al., 2018; Legrand et al., 2020). As recently reviewed by Kim et al. (2018), because of the tight association between UPS substrates and a wide variety of prevalent and severe diseases, UPS harbors great potential for drug targeting and therapeutic approaches, with tremendous potential impact on pharmaceutical intervention. Cancer, AIDS, cystic fibrosis, Alzheimer’s and Parkinson’s disease, and diabetes have all been found to be influenced by molecules secreted in a non-canonical fashion (Woodbury and Ikezu, 2014; Kim et al., 2016, 2018).

To study UPS in mammalian cells, different experimental setups have been explored. These include *in vivo* studies in mice (Dupont et al., 2011; Giuliani et al., 2011), *in vitro* studies on human cell cultures (Kim et al., 2016) on HeLa cells or on innovative artificial constructs such as inside-out vesicles as described in Schäfer et al. (2004), *in silico* analysis, and docking experiments to decipher secretion motifs and protein structure. In UPS *in vitro* studies, the chosen system and induction conditions are essential. The limitations lie in the fact that each treatment will disproportionately affect signaling pathways, leading to indirect effects (Giuliani et al., 2011).

Notably, even though much effort has been invested to elucidate UPS in mammalian cells, most molecular mechanisms remain unclear.

### Unconventional Protein Secretion in Protist Parasites

Proteins that find their way to the cell surface without entering the ER–Golgi pathway are also present in parasitic protozoa. When investigating secretomes of several protist parasites, researchers have found substrates that do not carry any known leader sequences but are either exposed on the cell surface or found in the extracellular medium. UPS has specifically been studied in some of the most prevalent parasitic genera (Table 2): *Leishmania* (Cuervo et al., 2009; MacLean et al., 2012), *Trypanosoma* (Geiger et al., 2010; Neves et al., 2014), *Plasmodium* (Möskes et al., 2004; Thavayogarajah et al., 2015), *Trichomonas* (Gómez-Arreaza et al., 2014; Miranda-Ozuna et al., 2016), and *Giardia* (Eckmann et al., 2000; Ringqvist et al., 2008; Touz et al., 2008; Skarin et al., 2011). UPS cargo often presents moonlighting functions when transported to the extracellular space (Jeffery, 1999). Furthermore, it has been suggested that moonlighting and UPS are directly linked to each other, as posttranslational modifications seem to influence function as well as transport of a protein (Tristan et al., 2011). Especially in glycolytic enzymes, proteins that do not carry a leader sequence for secretion, moonlighting of extracellular proteins is strikingly common (Jeffery, 1999; Collingridge et al., 2010; Gómez-Arreaza et al., 2014). The diverse functions of these unconventionally translocated proteins, essential for the invasion and persistence of parasites, suggest that glycolytic enzymes may be among the most important UPS cargos in protist parasites.

**TABLE 2:** Overview of selected parasitic UPS cargo, including parasitic species and evidence for moonlighting functions.

**Selected parasitic UPS cargo**	**Function**	**Selected studied organism/s**	**Evidence for functional moonlighting**	**Selected references**
Enolase	Glycolytic enzyme, virulence factor	*Giardia*, *Entamoeba*, *Plasmodium*, *Trichomonas*	Yes	Tovy et al., 2010; Ghosh et al., 2011; Miura et al., 2012; Ahn et al., 2018
Triosephosphate isomerase (TvTIM)	Glycolytic enzyme, virulence factor	*Trichomonas*	Yes	Miranda-Ozuna et al., 2016
Glyceraldehyde-3-phosphate dehydrogenase (GAPDH)	Glycolytic enzyme, virulence factor	*Entamoeba*, *Trypanosoma*, *Trichomonas*, *Plasmodium*	Yes	Grébaut et al., 2009; Lama et al., 2009; Biller et al., 2014; Cha et al., 2016; Miranda-Ozuna et al., 2016
Translation elongation factor 1 alpha (TEF-1α)	Elongation factor, virulence factor	*Plasmodium*, *Leishmania*, *Trypanosoma*, *Giardia*	Yes	Nandan et al., 2002; Silverman et al., 2008; Grébaut et al., 2009; Skarin et al., 2011; Demarta-Gatsi et al., 2019
Hydrophilic acylated surface protein B (HASPB)	Parasite transmission, virulence factor	*Leishmania*	No	MacLean et al., 2012; Kim et al., 2018

### Leishmania and *Trypanosoma*

As Silverman et al. (2008) have shown, secretion in *Leishmania* seems to involve UPS on a regular basis. The secretome analysis of *Leishmania donovani* revealed that the majority of secreted proteins do not carry leader sequences and must, therefore, be secreted unconventionally (Silverman et al., 2008). The same seems to be true for a close relative of *Leishmania*, as *Trypanosoma* has been suggested to employ UPS substantially (Geiger et al., 2010; Neves et al., 2014). Geiger et al. (2010) could demonstrate that only a small portion of secreted proteins carry secretion motives. It was proposed that proteins might be secreted via microvesicles, budding off at an organelle called the flagellar pocket (Geiger et al., 2010). In *Leishmania* as well as in *Trypanosoma*, there is clear evidence that UPS plays a crucial role in these species’ virulence (Silverman et al., 2008; Geiger et al., 2010). Nevertheless, further insight into secretion machineries is still needed.

A relatively well-studied UPS substrate is translation elongation factor 1 alpha (TEF-1α). TEF-1α was found in the secretomes of *Trypanosomes* (Grébaut et al., 2009) as well as in *Leishmania* (Silverman et al., 2008). *Leishmania*-derived TEF-1α leads to deactivation of host macrophages (Nandan et al., 2002) and was used in an anti-leishmanial vaccine LeishDNAvax, as one of several antigens (Das et al., 2014; Naouar et al., 2016). Even though in *Leishmania* the secretion mechanism of TEF-1α has not yet been elucidated, this protein presents no classical secretory motifs in its amino acid sequence (Nandan et al., 2002). Another well studied translocation is the unconventional secretion of HASPB (hydrophilic acylated surface protein B) in *Leishmania* species. The function of HASPB is not fully elucidated, but it is suggested that it is involved in parasite transmission from the sand fly to the mammalian host or in establishing the parasite in the host macrophages (MacLean et al., 2012; Kim et al., 2018). The molecular machinery behind the secretion of HASPB is only partly understood. Rather than being transported by intracellular vesicles (Nickel, 2005), HASPB export involves ABC transporter-mediated processes, in a UPS type II route (Denny et al., 2000; Stegmayer et al., 2005; MacLean et al., 2012; Kim et al., 2018). While not carrying a classic secretory signal sequence, the N-terminal myristoylation of this protein is suggested to direct HASPB to the plasma membrane (Denny et al., 2000).

This N-terminal domain is necessary and sufficient since HASPB expressed heterologously in mammalian cells is correctly secreted, suggesting a highly conserved export machinery, which allows the export signal to be recognized by higher eukaryotes (Denny et al., 2000).

The glycolytic enzyme and widely studied UPS cargo GAPDH (glyceraldehyde-3-phosphate dehydrogenase) was found in *Trypanosoma* exosomes (Geiger et al., 2010). In the secretome identification study of Grébaut et al. (2009), it was pointed out that, being involved in energy metabolism of the parasite, GAPDH is a virulence factor in Trypanosomes and a potential drug target (Grébaut et al., 2009).

### Plasmodium

In the malaria parasite *Plasmodium*, protein secretion has been intensively studied because of its complexity due to several membranes a parasite protein has to travel through (Möskes et al., 2004). There are, for example proteins, that are targeted to the hosts red blood cells and have to be transported past the own cell membrane, then the parasitophorous vacuole membrane before even reaching the host cell. The unconventional secretion of enolase, a glycolytic enzyme, has been studied in a wide range of organisms. As experiments in yeast suggest, unconventional secretion might often be mediated by SNARE proteins (Miura et al., 2012). In *Plasmodium*, enolase seems to be of high importance for the parasite in the mosquito stage, while the secretion mechanisms are unclear (Ghosh et al., 2011). Ghosh et al. (2011) have studied *Plasmodium* ookinetes and found that enolase lines the ookinete surface and plays a crucial role in midgut invasion (Ghosh et al., 2011). While acting as an interaction ligand to the midgut epithelium of the mosquito, it also captures plasminogen, which in turn promotes the invasion machinery, both functions ultimately promoting the cell invasion process (Ghosh et al., 2011).

As mentioned for trypanosomes, GAPDH, is an extracellular glycolytic enzyme similarly widespread in parasites as enolase (Gómez-Arreaza et al., 2014). In *Plasmodium*, it plays a role in vesicular transport and host cell invasion (Daubenberger et al., 2003; Cha et al., 2016). It was found that GAPDH is located on the surface of *Plasmodium* sporozoites and mediates parasite traversal through Kupffer cells and infection of liver cells, therefore, performing a crucial role as virulence factor of this parasite (Cha et al., 2016). *Plasmodium* GAPDH is suggested as a promising vaccine candidate for a prehepatic vaccine antigen, as the sequence of *Plasmodium* GAPDH seems sufficiently different from mammalian GAPDH (Cha et al., 2016).

A protein not belonging to the group of glycolytic enzymes, which gained just as much interest in *Plasmodium* research as it has in Trypanosomes, is TEF-1α (Demarta-Gatsi et al., 2019). TEF-1α is another good example of an unconventionally secreted moonlighting protein. As a study on *Plasmodium berghei* shows, in this parasite, TEF-1α is secreted via extracellular vesicles (Demarta-Gatsi et al., 2019). Plasmodia deficient in TEF-1α was no longer able to suppress T-cell response (Demarta-Gatsi et al., 2019). Furthermore, immunization of the host with this protein led to long-lasting protection from the parasite (Demarta-Gatsi et al., 2019).

The *Plasmodium falciparum* adenylate kinase 2 (PfAK2) secretion pathway shows a similar pattern to what was found in HASPB secretion in *Leishmania*, highlighting the importance of cotranslational modifications in targeting proteins for unconventional secretion pathways (Denny et al., 2000; Nickel, 2005; Thavayogarajah et al., 2015). Similar to HASPB, three N-terminal motifs found in PfAK2 were sufficient to target and translocate this protein across the parasite plasma membrane, an N-terminal myristoylation, a putative palmitoylation, and several basic amino acid residues in the 37 terminal amino acids (Thavayogarajah et al., 2015).

We attempted to reconstruct and compare the three-dimensional structure of the N-terminal 37 amino acids of PfAK2 as well as the N-terminal 55 amino acids of *Leishmania* HASPB using the Robetta web server.^1^ Even though both modeled N-termini showed large helical structures, the obtained models were either low confidence ones (e-values of 0.41 for PfAK2, 0.28 for HASPB) in comparative modeling (Song et al., 2013) or presented very high angstrom error estimates using the TrRefineRosetta modeling method (Hiranuma et al., 2021; Yang et al., 2020). α-Helical structures are typical for classic secretory signal peptides (Emr and Silhavy, 1983); if present in UPS substrates, they may mimic membrane-association domains, which may favor membrane traversal.

### Trichomonas

The sexually transmitted parasite *Trichomonas vaginalis* secretes virulence factors to the cell surface to adhere to the vaginal and cervical epithelia to be able to establish in this difficult microenvironment (Miranda-Ozuna et al., 2016). Several of these virulence factors have been studied for their secretion. TvTIM (triosephosphate isomerase) has been found on the surface of the parasite in addition to TvENO and seems to be an important part of host–pathogen interactions for this parasite (Miranda-Ozuna et al., 2016). TvTIM was studied specifically for its secretion pathways, and it was found that it might employ more than one route of UPS. In localization assays performed by Miranda-Ozuna et al. (2016), this protein was shown to be unconventionally secreted either via vesicles, or free of cytoplasmic vesicles, while the detailed underlying mechanism involved in its secretion could not be unraveled. TvGAPDH too is located on the cell surface and acts as a fibronectin-binding protein, being a crucial factor in host colonization and persistence (Lama et al., 2009; Miranda-Ozuna et al., 2016).

### Giardia and *Entamoeba*

*Giardia lamblia* is an especially interesting organism when studying unconventional protein secretion. This organism lacks organellar structures reminiscent of a Golgi apparatus and is, therefore, an ideal system to study protein secretion independently of a classical ER–Golgi route (Palade, 1975; Rothman, 1994). The pathogenesis of this parasite is yet to be fully unraveled. During infection, *Giardia* trophozoites attach tightly to the intestinal walls of their host, but remain extracellular throughout their life cycle. The host experiences various symptoms such as nausea, vomiting, epigastric pain, and diarrhea (Ankarklev et al., 2010). Nutrient depletion of the host, caused by the feeding of *Giardia*, as well as secreted cargo by the parasite might be reasons for these symptoms. When comparing secretion in axenic cultures of *G. lamblia* trophozoites to cocultures with intestinal epithelial cells, giardial enolase was a protein that was considerably enriched in the medium of cocultured cells, suggesting that epithelial cells must have triggered enhanced *Giardia* enolase secretion in this model system (Eckmann et al., 2000; Ringqvist et al., 2008). Parasite-derived enolase, ornithine carbamoyltransferase (OCT), TEF-1 α, and arginine deiminase (ADI) were all found extracellularly in the absence of a signal sequence (Eckmann et al., 2000; Ringqvist et al., 2008; Touz et al., 2008; Skarin et al., 2011). The capability of enolase to show moonlighting functions is demonstrated in the intestinal parasite *Entamoeba histolytica*, where enolase shows a cytosolic as well as a nuclear localization (Tovy et al., 2010). In the nucleus, it interacts with, and regulates, Dnmt2 (cytosine-5 methyltransferase 2) activity, which catalyzes DNA methylation, whereas when secreted, enolase potentially functions as a virulence factor (Riahi et al., 2004; Tovy et al., 2010; Ahn et al., 2018). In *E. histolytica*, GAPDH seems likely to be another unconventionally secreted virulence factor playing a role in colonization of the host, influencing adhesion (Biller et al., 2014).

The role of ADI and OCT in *G. lamblia* host–pathogen interaction was specifically investigated in connection to local arginine depletion in the gut, as arginine depletion has a negative impact on host cell-dependent nitric oxide production and promotes parasite adhesion to the intestinal epithelium (Stadelmann et al., 2012; Banik et al., 2013). Alongside virulence factors, *G. lamblia* cells secrete large amounts of membrane-anchored variant surface antigens involved in antigenic variation for immune evasion (Gargantini et al., 2016). These variant surface proteins (VSPs) carry a leader sequence; however, since *Giardia* does not contain a Golgi apparatus, VSP secretion is effectively a Golgi bypass, UPS pathway type IV trafficking route (Gargantini et al., 2016). Secretome and surface proteome analysis of *Giardia* have revealed a large number of proteins, which are translocated across the plasma membrane without carrying a signal sequence, making these cargos highly interesting for studies on UPS machinery (Ma’ayeh et al., 2017; Dubourg et al., 2018; Davids et al., 2019).

## Open Questions and Future Perspectives

Even though the first secreted proteins without a classical signal sequence for secretion have been known for over three decades (Auron et al., 1984), the potential impact and benefits of deeper knowledge on UPS only just recently become more evident. Many open questions remain concerning the molecular mechanisms of translocation of most UPS cargos. In protist parasites, higher accessibility to whole secretomes and surface proteomes led to the discovery of a large number of putative UPS cargos (Silverman et al., 2008; Grébaut et al., 2009; Lama et al., 2009; Geiger et al., 2010; Biller et al., 2014; Cha et al., 2016; Miranda-Ozuna et al., 2016). Even though categorization of UPS substrates according to their secretion pathway has been attempted (Rabouille, 2017), a lack of knowledge on the variety and details of molecular translocation machineries make it difficult to find meaningful categories, which can be generally applied, as the example of IL-1β shows.

There are still many open questions, which will have to be solved for every cargo individually. How do proteins without a classic signal sequence enter a translocation pathway; what singles them out? Are there “unconventional” signal sequences for secretion? To solve these questions, what is sorely needed are *ad hoc in silico* prediction tools. Using an *in silico* program for sequence-based UPS prediction, such as SecretomeP, may aid the investigation of cargos of interest when studying unconventional secretion. Nevertheless, it is important to note that the algorithms in SecretomeP are trained in mammalian and bacterial proteins, thus, limiting the reliability of predictions in other lineages (Bendtsen et al., 2004; Lonsdale et al., 2016). Another set of questions should be asked concerning the evolution of unconventional secretion. UPS appears to be a widely distributed phenomenon across the evolutionary tree of life. Considering how many substrates, lineages, and mechanisms are involved, UPS may have evolved many times independently, although some pathways may be conserved. With regard to UPS cargo, the secondary structure of the cargos might play an even more important role here than peptide sequence *per se*. Might there be structural motifs that are preserved, acting as signals for secretion? Working toward answers for all these questions might turn out to be highly beneficial for translational research with UPS as a target for drug and vaccine development (Kim et al., 2018).

## Conclusion

The topic of UPS and its related mechanisms is gaining more and more interest in the field of protein research in protist parasites. One fundamental difference in unconventional secretion between mammalian cells and parasitic protists is that protist parasite cells secrete certain substrates unconventionally in normal growth conditions. In contrast, UPS in mammalian cells seems most often to be triggered by cellular stress, inflammation, starvation, ER stress, or mechanical stressors (Giuliani et al., 2011; Merani et al., 2015; Kim et al., 2016; Rabouille, 2017). The reason why mammalian cells are employing UPS when the cell is under a severe stressor is not fully understood. The strong connection in mammalian UPS to immune response and inflammation processes makes it likely that substrates of UPS have key roles for survival of the organism. When impaired, severe illness or death could be a likely outcome. Therefore, from an evolutionary point of view, the reduction in complexity of the pathway might be under strong selective pressure, while under normal conditions, there is no pressure to streamline the secretion process. Interesting findings in neuronal research suggest that Golgi bypassing, UPS type IV, is not only widespread in several neuron cell types but also affects a large amount of key membrane proteins on a regular basis, while the cells are not under stress conditions (Hanus et al., 2016; Kennedy and Hanus, 2019). This bypass, or the core glycosylation connected to the studied bypass, leads to the affected proteins being unusually fragile (Hanus et al., 2016). This instability reduces the distance the proteins are able to travel before degrading, limiting them to exploring synapses very locally (Hanus et al., 2016). It is possible that UPS in this case is a way of fine tuning the release of key membrane proteins, making them only available to few synapses for a short period of time (Hanus et al., 2016; Kennedy and Hanus, 2019). This example shows that certain mammalian cell types are employing UPS without the influence of stressors and suggests that UPS pathways are much more regularly used than initially thought.

Parasites are well known in evolutionary biology for being a good example of reductive evolution. The parasitic lifestyle allows these organisms to reduce certain metabolic pathways or even organelles, wherever the function can be replaced or hitchhiked from its host organism, making parasites very efficient life forms with the main focus of their cell functions to persist, reproduce, and spread to new hosts. The environmental changes a parasite faces urges the organism to adapt as quickly as possible to the new habitat as, if it fails to do so, it will have fatal consequences, and the parasite will not be able to establish in a host or its vector (Ginger, 2014). This is in line with the selection pressure to reduce complexity of protein secretion in parasitic protists.

Another advantage of unconventional secretion pathways is that, with separate pathways, the cell prevents unwanted interactions, for example, between glycoproteins and their ligands at the wrong time or location (Popa et al., 2018). While the ER–Golgi-dependent secretion pathway seems to be advantageous for the majority of proteins in mammalian cells under normal circumstances, UPS as a streamlined alternative seems to bring a selective advantage in a parasitic lifestyle and in mammalian cells under stress. Furthermore, many UPS studies concern cargos displaying moonlighting functions, suggesting that UPS and moonlighting are closely connected (Jeffery, 2018). Both unconventional secretion pathways as well as moonlighting functions of a protein are parsimonious changes to their classic alternatives and, therefore, represent a streamlining of this essential cellular process.

A better understanding of underlying secretion mechanisms could reveal possible ways to regulate or prevent export of UPS substrates involved in an astonishing variety of health and disease-related pathways. The fact that UPS plays an essential role in some of the most prevalent, most life-threatening diseases, points out the significance of research on UPS to understand these unconventional pathways, which seem not so unconventional after all.

## Author Contributions

EB produced the illustration. Both authors wrote and revised the manuscript.

## Conflict of Interest

The authors declare that the research was conducted in the absence of any commercial or financial relationships that could be construed as a potential conflict of interest.

## Footnotes

1 https://robetta.bakerlab.org/
